# HIF1*α*: A Novel Biomarker with Potential Prognostic and Immunotherapy in Pan-cancer

**DOI:** 10.1155/2022/1246267

**Published:** 2022-07-08

**Authors:** Yonggang Tian, Feihu Bai, Dekui Zhang

**Affiliations:** ^1^Department of Gastroenterology, Lanzhou University Second Hospital, Lanzhou, Gansu Province, China; ^2^The Gastroenterology Clinical Medical Center of Hainan Province, Department of Gastroenterology, The Second Affiliated Hospital of Hainan Medical University, Haikou, China

## Abstract

Cancer is a catastrophic disease that seriously affects human health. HIF1*α* plays an important role in cancer initiation, progression, and prognosis. However, little is known about the specific role of HIF1*α* in pan-cancer. Therefore, we systematically and comprehensively analyzed HIF1*α* using GEPIA, HPA, GeneMANIA, STRING, SMPDB, cBioPortal, UALCAN, and TISDB databases and also 33 cancer and normal tissues in TCGA downloaded from the Genome Data Commons (GDC) data portal. Data and statistical analysis were performed using R software v4.0.3. Our results found that there were differences in the mRNA expression levels of HIF1*α* in human pan-cancer and its corresponding normal tissues. The expression level of HIF1*α* correlated with tumor stage in LIHC and also significantly correlated with prognosis in LIHC, LUSC, STAD, OV, PAAD, PRAD, THCA, LUAD, MESO, and READ. The small molecule pathways involved in HIF1*α* include succinate signaling, fumarate, and succinate carcinogenesis-related pathways. The highest mutation frequency of the HIF1*α* gene in pan-cancer was head and neck cancer, and the HIF1*α* methylation level in most tumors is significantly reduced. HIF1*α* was not only associated with immune cell infiltration but also with immune checkpoint genes and immune regulators TMB and MSI. There were currently 5 small molecule drugs targeting HIF1*α*.

## 1. Introduction

As the socioeconomic status and access to health care improve, the disease burden of the population tends to shift epidemiologically: the population appears to have transitioned from contracting primarily communicable diseases to developing primarily noncommunicable diseases [1]. Cancer is a devastating disease among noncommunicable diseases. With an estimated 19.3 million new cancer cases and nearly 10 million cancer deaths worldwide in 2020, the global cancer burden is expected to reach 28.4 million in 2040, a 47% increase from 2020 [2]. Although new technologies and new drugs for the prevention and treatment of cancer are constantly emerging, the prevention and treatment of cancer still cannot meet the needs of the growing number of cancer patients. Therefore, there is still a long way to actively search for specific and sensitive biomarkers for cancer prevention and to develop new drugs.

HIF-1 (hypoxia-inducible factor-1) was first discovered by Semenza and Wang in 1992, and then the structure of HIF-1 was established and the coding sequence of its cDNA was proved [3, 4]. HIF-1 is ubiquitously present in human and mammalian cells and is also expressed under normoxia (21% O_2_). The study found that the median oxygen level in most tumors was <2%. Therefore, HIF-1 is often expressed in tumors [5]. Moreover, an increasing number of studies have found that HIF-1 can be involved in metabolic reprogramming [6–8], angiogenesis [7, 9, 10], stem cell [10], and immune regulation [11, 12]. Additionally, HIF-1 activity is associated with increased cancer mortality [13], invasion [14, 15], metastasis [15, 16], immune evasion [17], and resistance to therapy [18–20], thus providing a rationale for the therapeutic targeting of these transcription factors in cancer [21, 22].

The study found that HIF1*α* is an important part of HIF-1 activity, and it consists of four parts: bHLH domain, PAS domain, ODD domain, and transactivation domain. Binding of the ODD domain to pVHL protein under hypoxic level can prevent HIF1*α* subunit ubiquitination and degradation, thereby increasing the expression level of HIF1*α* protein, and tumors adapt to the hypoxic environment by expressing high levels of HIF1*α* protein [23]. To the best of our knowledge, a comprehensive analysis of HIF1*α* on human pan-cancer clinical prognosis, immune microenvironment, and HIF1*α*-targeted drugs using bioinformatics remains largely unknown. Herein, we use bioinformatics to comprehensively and systematically study the role of HIF1*α* in human pan-cancer. Our results suggest that the expression of HIF1*α* is related to tumor prognosis and immune cell infiltration. In addition, our study also provides information on the involvement of HIF1*α* in signaling pathways and current drugs targeting HIF1*α*. In summary, these findings provide insights into the growing interest in HIF1*α* between the diagnosis and treatment of cancer.

## 2. Materials and Methods

### 2.1. GEPIA Database

GEPIA (http://gepia.cancer-pku.cn/) is a web-based tool that provides fast and customizable functionality based on TCGA and GTEx data [24]. In this study, we used the GEPIA database to analyze the expression of HIF1*α* in tumor tissues and their corresponding normal tissues and displayed them using BodyMap and dot plot, respectively. Subsequently, we also used this database to explore the correlation between HIF1*α* expression and tumor pathological stage. All of the above use log2(TPM+1) for log scale. In addition, we used the “Survival Plots” module to explore the relationship between HIF1*α* expression and pan-cancer prognosis.

### 2.2. HPA Database

HPA (https://www.proteinatlas.org/) database is a large-scale initiative to map the entire human proteome using the integration of antibody-based proteomics and various other omics techniques [25]. In our study, we explored the mRNA expression levels of HIF1*α* in human cell lines based on the HPA database; the gene expression levels are represented as log2 TPM values.

### 2.3. GeneMANIA Database

GeneMANIA (http://genemania.org) analyzes association data including protein and genetic interactions, pathways, coexpression, colocalization, and protein domain similarity [26]. We, in this study, explored the protein-protein interaction network of HIF1*α* using this database.

### 2.4. STRING Database

STRING (https://cn.string-db.org/) enables the analysis of sources of protein-protein interaction information [27]. This database was also used in our study to explore the protein-protein interaction network of HIF1*α*.

### 2.5. SMPDB

SMPDB (https://smpdb.ca/) is a comprehensive, colorful, fully searchable, and highly interactive database for visualizing human metabolism, drug action, drug metabolism, physiological activity, and metabolic disease pathways [28, 29]. In this study, we used this database to explore the small molecule pathways involved in HIF1*α*.

### 2.6. cBioPortal Database

cBioPortal (http://www.cbioportal.org), which provides a web resource for exploring, visualizing, and analyzing multidimensional cancer genomic data [30], was used to explore the HIF1*α*. 2922 total samples (including 2583 patients) (CGC/TCGA, Nature 2020) were analyzed. mRNA expression *z* scores (RNA Seq V2 RSEM) were obtained using a *z* score threshold of ±2.0.

### 2.7. UALCAN Database

UALCAN (http://ualcan.path.uab.edu/index.html) database enables genomics, bioinformatics, and integrative approaches to understand the molecular basis of cancer [31]. We, in our present work, investigated HIF1*α* methylation levels in pan-cancer and its corresponding normal tissues based on the UALCAN database. The significance of differences was evaluated using Student's *t*-test, and *p* < 0.05 was considered statistically significant.

### 2.8. TISDB Database

TISDB (http://cis.hku.hk/TISIDB/) database is a user-friendly portal that integrates multiple types of data resources in tumor immunology [32]. In our study, the TISDB database was used for the analysis of drugs targeting HIF1*α*.

### 2.9. HIF1*α* Expression Level and Prognosis of Tumor Patients

The data of 33 types of cancer and normal tissues in the TCGA dataset (https://portal.gdc.com) were downloaded from the Genomic Data Commons (GDC) data portal website, using the univariate Cox regression analysis, and the forest was used to show the *p* value, HR, and 95% CI of each variable through “forest plot” R package. All the analysis methods and R package were implemented by R version 4.0.3. Two-group data was performed by the Wilcoxon test. *p* < 0.05 were considered statistically significant.

### 2.10. HIF1*α* Expression and Immune Cell Infiltration and Immune Modulator Genes

The data of 33 types of cancer and normal tissues in TCGA were downloaded from the Genomic Data Commons (GDC) data portal website. We obtained immune scores using an R package “Immunedeconv” that integrates two state-of-the-art algorithms, including TIMER and xCell. The Spearman correlation analysis heatmaps of HIF1*α* gene expression and genes associated with immune scores or immune checkpoints in different types of cancers were generated, the vertical axis represents different immune scores, and different colors represent correlation coefficients. R software v4.0.3 was used for statistical analysis. *p* < 0.05 were considered statistically significant.

### 2.11. Pan-cancer Analysis of the Correlation between HIF1*α* Expression and Immune Regulators TMB and MSI

We obtained TMB and MSI scores from the dataset downloaded from TCGA using R software v4.0.3 for statistical analysis and Spearman correlation analysis of TMB, MSI, and HIF1*α* gene expression. The abscissa represents the correlation coefficient between genes and TMB, and the ordinate represents different tumors. The size of the dots represents the size of the correlation coefficient, and different colors represent the significance of the *p* value. The bluer the color, the smaller the *p* value.

## 3. Results

### 3.1. The mRNA Expression Landscape of HIF1*α* in Human Pan-cancer

**Figure 1:**
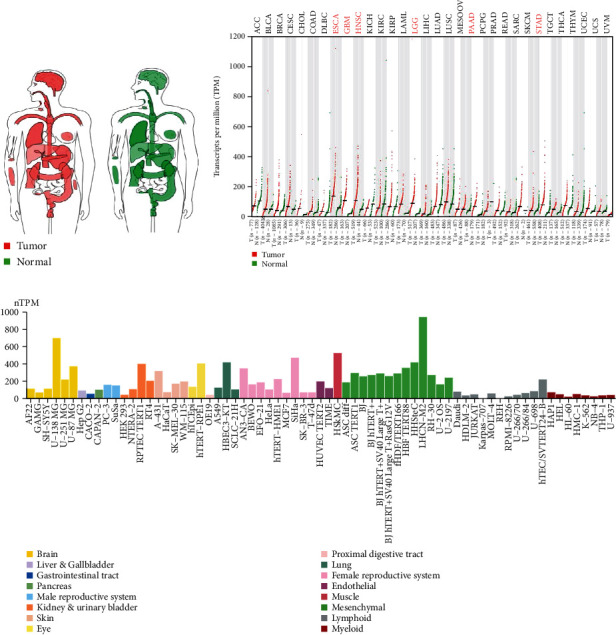
The mRNA expression landscape of HIF1A in human pan-cancer. (a) Interactive BodyMap and (b) dot plot, the HIF1A median expression of tumor and normal samples in BodyMap from GEPIA. Each dot represents expression of samples. (c) The mRNA expression levels of HIF1A in cell lines based on HPA database.

To explore the mRNA expression landscape of HIF1*α* in human pan-cancer, we comprehensively analyzed the mRNA expression levels of HIF1*α* in interactive body maps using the GEPIA dataset. We know that, from the overall level of the interactive body map, the median expression levels of HIF1*α* in most human tumor tissues and their corresponding normal tissues are different, in particular in the brain, blood, lungs, digestive organs (esophagus, pancreas, stomach, and gallbladder), kidneys, thyroid, and other tissues and organs (Figure 1(a)). Based on the previous findings, we, next, studied the mRNA expression levels of HIF1*α* in 33 tumors and their corresponding normal tissues using the GEPIA database. Unexpectedly, the median level of mRNA expression of HIF1*α* was high in only 7 tumor tissues (ESCA, GBM, HNSC, LAML, LGG, PAAD, and STAD) compared to normal tissues (Figure 1(b)). Finally, we further analyzed the mRNA expression level of HIF1*α* from the cellular level using the HPA database. As a result, we found higher levels of HIF1*α* mRNA expression in these tissue organ cell lines, including the brain, liver and gallbladder, gastrointestinal pancreas, male reproductive system, kidneys and bladder, skin, eyes, proximal gastrointestinal, lungs, female reproductive system, endothelial, muscle, and mesenchymal lymphoid myeloid (Figure 1(c)). Regardless of whether tumor tissue is compared with its corresponding normal tissue, or at the level of organ cell lines, the mRNA expression levels of HIF1*α* are higher in organs like the brain, digestive organs (esophagus, pancreas, stomach, and gallbladder), and lungs.

### 3.2. Correlations between the HIF1*α* Expression and Tumor Pathological Stage

**Figure 2:**
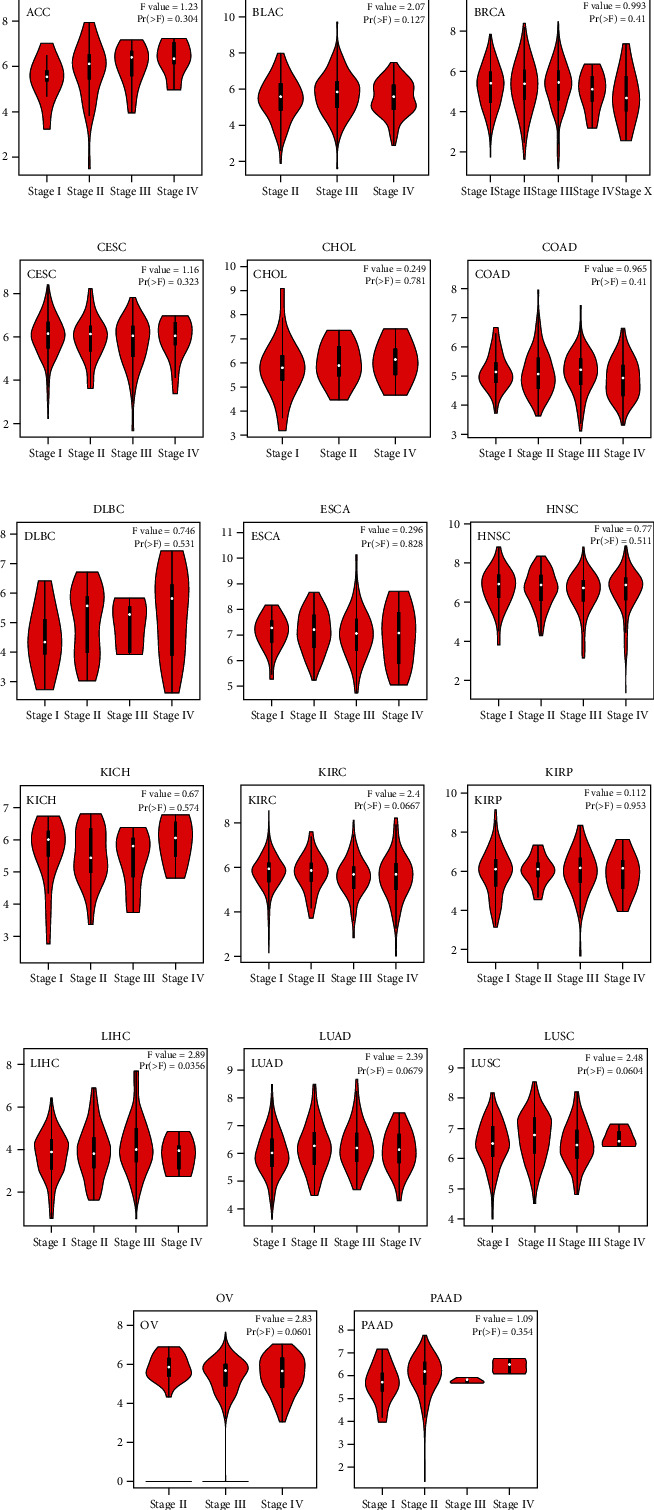
Correlations between the HIF1A expression and tumor including (a) ACC, (b) BLAC, (c) BRCA, (d) CESC, (e) CHOL, (f) COAD, (g) DLBC, (h) ESCA, (i) HNHC, (j) KICH, (k) KIRC, (l) KIRP, (m) LIHC, (n) LUAD, (o) LUSC, (p) OV, and (q) PAAD pathological stage from GEPIA. Log2 (TPM+1) was used for log scale.

As one of the important indicators of patient prognosis, the pathological stage of the tumor should be looked at closely. Hence, we used the GEPIA dataset to analyze the correlation between HIF1*α* expression level and tumor pathological stage, including 17 tumors, while other tumors could not be shown in the GEPIA database. As a complete surprise, our results showed that HIF1*α* expression was only significantly correlated with tumor stage in LIHC (*p* = 0.0356), and there was no correlation between the expression level of HIF1*α* and the pathological stage of other tumors (*p* > 0.05) (Figure 2). Collectively, these results demonstrate that the expression level of HIF1*α* is associated with the pathological staging of LIHC, which has a certain guiding significance for guiding the pathological staging of this tumor.

### 3.3. The Relationship between HIF1*α* Expression Level and Prognosis of Tumor Patients

**Figure 3:**
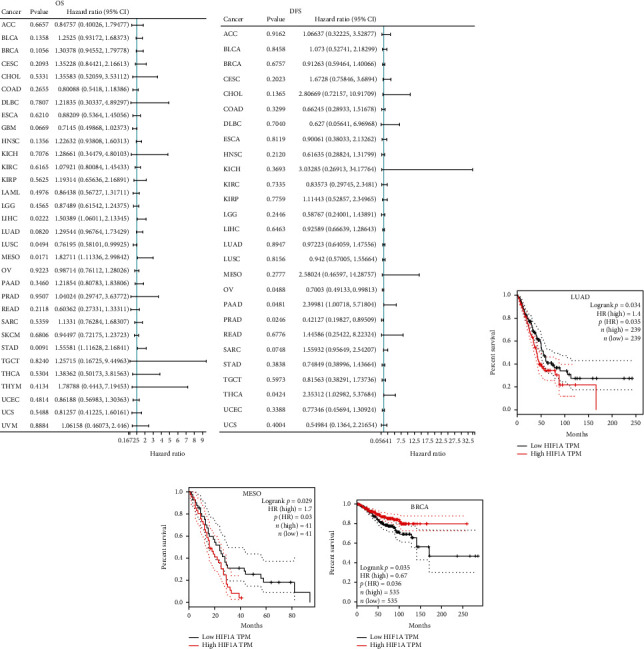
Association between HIF1A expression and prognosis in cancer patients. (a and b) A forest plot of hazard ratios of HIF1A in 33 types of tumors. (c and d) Kaplan-Meier survival curves of OS for patients stratified by the different expressions of HIF1A in LUAD and MESO. (e) Kaplan-Meier survival curves of DFS for patients stratified by the different expressions of HIF1A in BRCA.

Based on the findings of HIF1*α* expression level and tumor pathological stage, we further evaluated the relationship between HIF1*α* expression level and survival prognosis of pan-cancer patients by the Cox regression analysis. Of note, the prognostic indicators in this study mainly include OS and DFS. The Cox regression analysis of the results from 33 types of cancer suggests that the expression level of HIF1*α* was significantly associated with OS in LIHC, LUSC, MESO, and STAD patients (*p* < 0.05) (Figure 3(a)). In addition, we also found that the expression level of HIF1*α* was significantly associated with DFS in 4 tumors, including OV, PAAD, PRAD, and THCA (*p* < 0.05) (Figure 3(b)). Next, we used the Kaplan-Meier survival curves to find that high expression of HIF1*α* in LUAD and MESO has worse OS (Figures 3(c) and 3(d)), while high expression of HIF1*α* in BRCA has better DFS (Figure 3(e)), and there was no statistical difference between the high and low expression of HIF1*α* in other tumors and OS and DFS (*p* > 0.05, supplementary Figure 1 and Supplementary Figure 2).

### 3.4. Protein-Protein Interaction Network and Small Molecule Pathway of HIF1*α*

**Figure 4:**
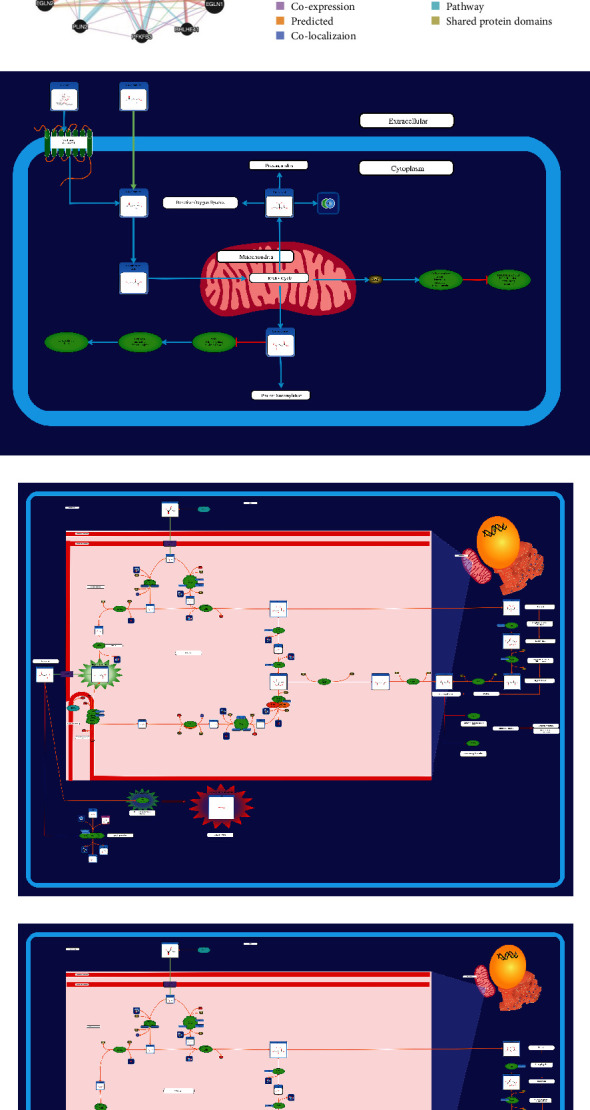
Protein-protein interaction network and functional enrichment of HIF1A. (a and b) Based on the GeneMANIA and STRING database to explore the protein-protein interaction network of HIF1A, respectively. (c–e) The use of SMPDB to analyze the small molecule pathway of HIF1A in humans.

To explore the protein-protein interaction network of HIF1*α*, our analysis using GeneMANIA databases found that there are 20 genes associated with HIF1*α* (Figure 4(a)). Furthermore, we explored using the STRING database and found that the number of nodes associated with HIF1*α* is 11 (Figure 4(b)). We used SMPDB analysis to find that HIF1*α* in human small molecule pathway includes one protein pathway, succinate signaling (Figure 4(c)), and two disease pathways, the oncogenic action of fumarate and the oncogenic action of succinate (Figures 4(d) and 4(e)).

### 3.5. Analysis of HIF1*α* Gene Mutation and Methylation Level in Pan-cancer

**Figure 5:**
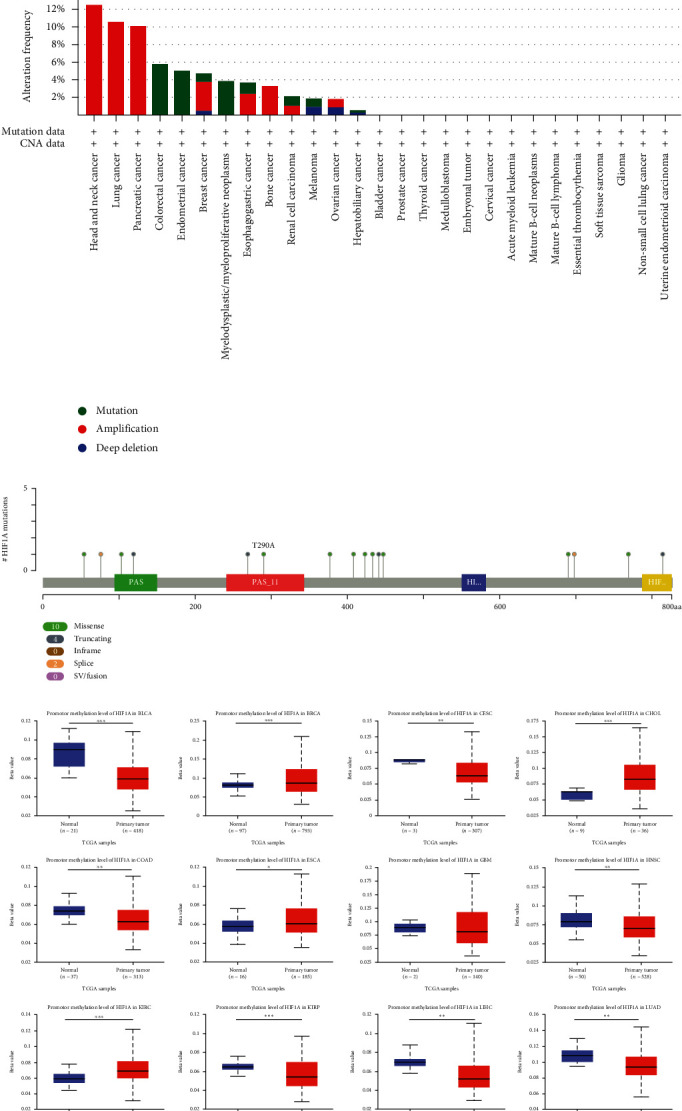
Analysis of HIF1A gene mutation and methylation level in pan-cancer. (a) The total mutations in the HIF1A gene were assessed using a genome-wide pancancer analysis in the cBioPortal database (ICGC/TCGA, Nature 2020). (b) The HIF1A gene alteration frequency with different types of mutations was examined using the cBioPortal database. (c) Mutation diagram of HIF1A in different cancer types across protein domains. (d) Analysis of HIF1A methylation levels in pancancer based on UALCAN database. ^∗^*p* < 0.05, ^∗∗^*p* < 0.01, and ^∗∗∗^*p* < 0.001.

To assess the mutation of HIF1*α* in pan-cancer, we conducted an in-depth study using the cBioPortal database and found that HIF1*α* was altered in 5% (132/2583) of pan-cancer patients (Figure 5(a)). In addition, we also analyzed the mutation frequency of the HIF1*α* gene in different types of tumors, and the results showed that the mutation frequency of head and neck cancer (12.5%), lung cancer (10.53%), and pancreatic cancer (10.06%) ranked the top three, respectively. Notably, amplification is the most common type of HIF1*α* gene mutation (Figure 5(b)). To better understand the mutational map of HIF1*α* in different cancer types across protein domains, our investigation found that a total of 16 mutation sites were detected, located between 0 and 826 (Figure 5(c)).

Aberrant DNA methylation is an important cause of cancer [33]. Hence, we next used the UALCAN database to explore the level of HIF1*α* methylation in pan-cancer and its corresponding tissues. Our results indicate that, compared with normal tissues, HIF1*α* methylation levels were significantly decreased in BLCA, BRCA, CHOL, CESC, COAD, ESCA, HNSC, KRIC, KIRP, LIHC, LUAD, LUSC, PAAD, READ, SARC, TGCA, and UCEC tissues (Figure 5(d)).

### 3.6. Pan-cancer Analysis of HIF1*α* Expression and Immune Cell Infiltration

**Figure 6:**
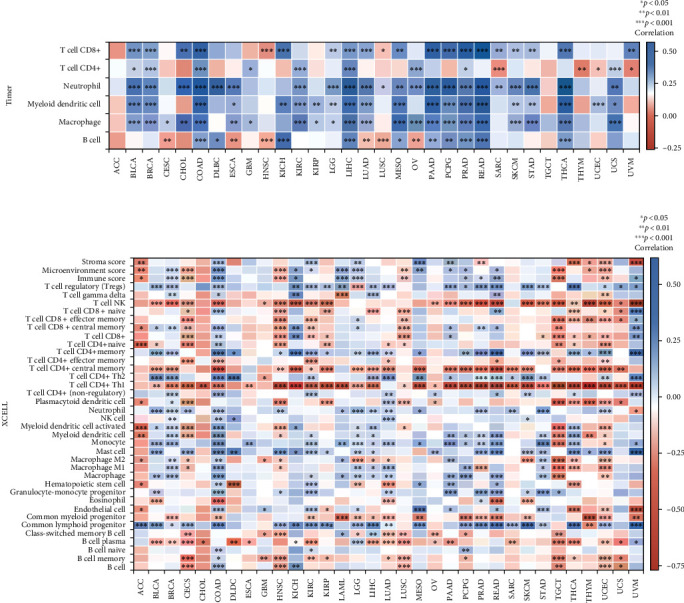
Pan-cancer analysis of HIF1A expression and immune cell infiltration. (a) The HIF1A expression significantly correlated with the infiltration levels of various immune cells in the TIMER database. (b) The HIF1A expression significantly correlated with the infiltration levels of various immune cells based on xCell. ^∗^*p* < 0.05, ^∗∗^*p* < 0.01, and ^∗∗∗^*p* < 0.001.

Since there is a certain relationship between HIF1*α* and immune response, we performed a pan-cancer analysis of the relationship between HIF1*α* expression and immune infiltration levels based on the TIMER database. The data presented here implicate that 20 cancers were associated with T cell CD8+, 12 cancers were associated with T cell CD4+, 22 cancers were associated with neutrophils, 20 cancers were associated with Myeloid dendritic cells, 22 cancers were associated with macrophage, and 16 cancers were associated with B cells (Figure 6(a)).

To further identify the relationship between HIF1*α* expression and infiltration of different types of immune cell subtypes, we used the xCell online tool to provide evidence that, among 38 immune cell subtypes, HIF1*α* expression was significantly negatively correlated with these subtypes of ACC, CESC, HNSC, KIRP, LUSC, TGCT, THYM, and UCEC, whereas HIF1*α* expression was significantly positively correlated with these subtypes of COAD, KICH, LAML, and LGG. Most remarkable, the expression of T cell CD4+ central memory, T cell CD4+ Th1, and HIF1*α* has the strongest negative correlation in various cancers (Figure 6(b)).

Together with the evidence presented here, this suggests that there is a certain correlation between HIF1*α* expression and the infiltration of various immune cells in the pan-cancer microenvironment.

### 3.7. Pan-cancer Analysis of the Correlation between HIF1*α* Expression and Immune Checkpoint Genes and Immune Regulators TMB and MSI

**Figure 7:**
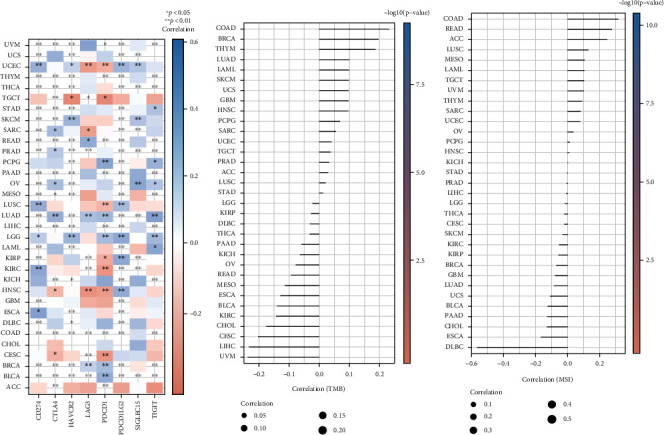
Pan-cancer analysis of the correlation between HIF1A expression and immune checkpoint genes and immune regulators TMB and MSI. (a) Pan-cancer analysis of the correlation between HIF1A expression and immune checkpoint genes. (b) Pan-cancer analysis of the correlation between HIF1A expression and immunomodulators TMB and MSI. ^∗^*p* < 0.05, ^∗∗^*p* < 0.01, and ^∗∗∗^*p* < 0.001.

To estimate the association between HIF1*α* expression and TME in the pan-cancer dataset, we further investigated the relationship between HIF1*α* expression and two major types of immune regulators. The vast majority of tumors include BLCA, BRCA, COAD, DLBC, ESCA, LAML, LIHC, LUAD, MESO, PAAD, PCPG, PRAD, READ, SARC, SKCM, STAD, THCA, THYM, UCEC, UCS, and UVM, and immune checkpoint genes are positively correlated. Only a few tumors including TGCT and HNSC were negatively correlated with immune checkpoint genes (Figure 7(a)).

TMB and MSI are two emerging biomarkers that are promising predictive biomarkers for immunotherapy in cancer treatment [34]. We first investigated the relationship between HIF1*α* expression and TMB. Our results suggest that the expression levels of HIF1*α* significantly correlated with TMB in COAD, BRCA, and LIHC (Figure 7(b)). In addition, in this report, we provide evidence that the expression level of HIF1*α* is significantly associated with MSI in some tumors, including COAD and DLBC (Figure 7(c)).

### 3.8. Drugs Targeting HIF1*α*

**Figure 8:**
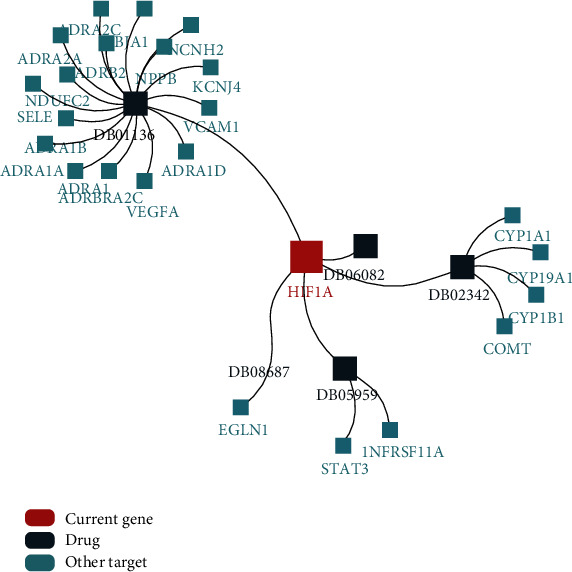
Drugs targeting HIF1A based on TISDB database. (a) Drugs targeting HIF1A collected from DrugBank database.

The development of drugs targeting HIF1*α* is critical for the treatment of cancer patients. Therefore, we used the TISDB database to analyze the current drugs targeting HIF1*α*. In the present study, we observed that there are 5 small molecule drugs targeting HIF1*α*, including carvedilol, 2-methoxyestradiol, ENMD-1198, PX-478, and FG-2216 (Figure 8(a)). Among them, carvedilol is the drug with the most targets, with a total of 17 targets. It downregulates HIF1*α* in the myocardium of volume-overloaded heart failure [35]. Remarkably, compared with carvedilol, the other 4 small molecule drugs involve relatively few targets. Of course, Table 1 presents more details on drugs targeting HIF1*α*.

## 4. Discussion

HIF is a central regulator for detecting and adapting to cellular oxygen levels, and it regulates oxygen homeostasis and metabolically activated genes through transcriptional activation. In addition to this, HIF affects many other processes including cancer development [9]. Current research suggests that HIF1*α*, as one of the most important members of the HIF family, is closely related to the occurrence, development, and prognosis of cancers [38–42]. Thus, we explored the role of HIF1*α* in human tumors by using bioinformatics methods. We first comprehensively analyze the expression of interacting BodyMap HIF1*α* in human tumors and their corresponding normal tissues. Second, we provide evidence that HIF1*α* expression levels are associated with the pathological staging of LIHC and that HIF1*α* expression levels are associated with prognosis in 11 tumors including LIHC, LUSC, MESO, STAD, OV, PAAD, PRAD, THCA, LUAD, MESO, and READ. In addition, we also found that HIF1*α* in human small molecule pathways includes 3 pathways (succinate signaling pathway, fumarate carcinogenesis, and succinate carcinogenesis), and HIF1*α* methylation levels are significantly reduced in most tumors. In addition to the above findings, we further discovered the relationship between HIF1*α* and immune cell infiltration in the cancer microenvironment and small molecule drugs targeting HIF1*α*.

In this study, our data suggest that the mRNA expression level of HIF1*α* was higher in organs such as the brain tumors, gastrointestinal tumors (esophagus, pancreas, stomach, and gallbladder), and lung tumors compared with normal tissues, both at the tissue level and the cell line level. Because we all know that the brain, as an advanced nerve center, needs a lot of oxygen and energy to maintain its normal function, the brain is most sensitive to hypoxia [43]. The stomach is an important digestive tract organ, and it is prone to stress-induced gastric ulcer during stress response [44], which is due to the sensitivity of gastric mucosa to ischemia and hypoxia. In addition to the aforementioned sensitivity to hypoxia, tumors grow faster and require more nutrients. Thus, cells like pancreatic cancer [45], liver cancer [46], and lung cancer [47] can activate the transcription of many genes, including those involved in energy metabolism, angiogenesis, and other protein products, by producing HIF1*α* to increase oxygen delivery or promote metabolic adaptation to hypoxia.

Next, we, on the one hand, found that the expression level of HIF1*α* was significantly correlated only with LIHC pathological stage, but not with other tumor stages. This finding may make HIF1*α* more clinically valuable as a new pathological staging marker for LIHC, but it would be interesting to further clarify the mechanism by which HIF1*α* is only associated with LIHC pathological staging. On the other hand, we provide evidence that, in pancancer, HIF1*α* expression levels were associated with LIHC, LUSC, MESO, STAD, OV, PAAD, PRAD, THCA, LUAD, and READ prognosis. Consistent with our findings, HIF1*α* expression levels are associated with poor prognosis in READ [48], LIHC [49], LUSC [50], MESO [51], STAD [52], OV [53], PAAD [41], PRAD [54], THCA [55], and LUAD [56]. How does HIF1*α* affect the prognosis of cancer patients? A few reports in the literature have documented that hypoxia is common in tumors. HIFA activity in hypoxic areas within often tumors mediates angiogenesis, epithelial-mesenchymal transition, stem cell maintenance, invasion, metastasis, and resistance to radiotherapy and chemotherapy [20, 57, 58].

We explored the protein-protein interaction network of HIF1*α* and found 20 genes associated with HIF1*α*. These HIF1*α*-related genes all play a role in cancer. For example, a study has found that HIF1*α* promotes EPO expression at the transcriptional level under hypoxia [59] and achieves antitumor effects by regulating Epo-activated signaling pathways as interfering with the cell cycle of brain tumors [60]. In addition, the HIF1*α*-related gene ENO1 can bind and degrade the expression of the hepcidin gene, thereby regulating the metabolic homeostasis of intracellular iron ions, affecting ferroptosis, and promoting the occurrence and development of liver cancer [61]. In conclusion, our discovery of HIF1*α*-related genes provides more new insights for diagnosis and treatment in pan-cancer. In addition, we also used SMPDB analysis to find that HIF1*α* in human small molecule pathways includes a protein pathway, the succinate signaling pathway, and two disease pathways, fumarate carcinogenesis and succinate carcinogenesis. Previous studies found that hydroxylase activity was inhibited in the presence of low concentrations of O_2_, high concentrations of tricarboxylic acid cycle intermediates (isocitrate, oxaloacetate, succinate, or fumarate), or chelating agents for Fe (II). The receptor for activated C-kinase 1 competes with heat shock protein 90 for binding to HIF-1*α* and mediates O_2_-dependent ubiquitination and proteasomal degradation [62].

Genetic errors in cancer cells reveal that fundamental biological processes required for cancer to develop and develop go awry. It became clear that cancer genomes contain many additional “passenger” mutations in the process. Driver and passenger DNA mutation patterns derived from cancer genomes provide clues to the different ways cancers manifest as genetically mutated diseases [63]. Interestingly, our data suggest that HIF1*α* has a mutation rate of 5% in pan-cancer. Therefore, in tumor diagnosis and treatment, some studies have developed HIF1*α* pharmacogenomic mutation models to study individual changes in the effects of tumor hypoxia drugs [64], which will guide precise treatment. Indeed, DNA methylation analysis is an emerging tool as an aid to improve the accuracy of pathological diagnosis; DNA methylation patterns in circulating tumor DNA hold great promise for minimally invasive cancer detection and classification [65]. We provide preliminary evidence that, according to the UALCAN database, HIF1*α* DNA methylation levels are reduced in 17 tumors. A study has confirmed that DNA hypomethylation activates gene transcription and increases tumor proliferation, migration, and metastasis [66]. Therefore, DNA hypomethylation predicts that these tumors tend to have a poor prognosis. In addition, a previous study demonstrated the involvement of HIF1*α* in the carcinogenesis of fumarate and succinate [67].

Enhancing immune cell function in tumors remains a major challenge in cancer immunotherapy. Hypoxia is a common feature of solid tumors, and cells adapt by upregulating the transcription factor HIF1*α* [40]. Our results indicate that most cancers are not only related to T cell CD8+, T cell CD4+, neutrophils, myeloid dendritic cells, macrophages, and B cells. And there is a certain relationship with the subtype of immune cells. For instance, our results suggest that, among 38 immune cell subtypes, HIF1*α* expression was significantly negatively correlated with these subtypes of ACC, CESC, HNSC, KIRP, LUSC, TGCT, THYM, and UCEC, whereas HIF1*α* expression was significantly correlated with these subtypes of COAD, KICH, LAML, and LGG. There was a significant positive correlation between subtypes. Among them, the expressions of T cell CD4+ central memory, T cell CD4+ Th1, and HIF1*α* have the strongest negative correlation in various cancers. Of course, a set of previous studies have also demonstrated that HIF1*α* expression is associated with infiltrating T cells and macrophages [68], Treg [69], and B cells [70].

Given the above findings, we next analyzed the relationship between HIF1*α* and immunomodulators and found that most tumors were positively associated with immune-checking genes. Only a few tumors, such as TGCT and HNSC, were inversely associated with immune check genes. In addition, we also explored the relationship between HIF1*α* and TMB and MSI and found that the expression level of HIF1*α* was significantly correlated with TMB in COAD, BRCA, and LIHC, and the expression level of HIF1*α* was correlated with MSI in COAD and DLBC. Of course, in addition to our study, a previous study confirmed that HIF1*α* expresses a new marker that separates the MSI-L group from the MSS and MSI-H groups [71]. In short, regardless of our findings, or those of previous studies, HIF1*α* is implicated in immune checkpoint genes, TMB, and MSI.

Finally, our study also investigated 5 small molecule drugs targeting HIF1*α*. Of these, carvedilol, a drug commonly used to treat high blood pressure, has recently been shown to protect the body from sunlight-induced cell damage and skin cancer [72]. Although the cancer preventive activity of carvedilol is independent of *β*-blockers, we envision whether the anticancer activity of carvedilol is related to HIF1*α*, which is a major topic for further research in the future.

In conclusion, in this study, our findings highly demonstrate the expression landscape of HIF1*α* in human pan-cancer and identify the relationship between HIF1*α* expression levels and tumor immune infiltration and HIF1*α*-targeting drugs. This may provide a new insight into the use of HIF1*α* to diagnose and treat human pan-cancer.

## 5. Conclusion

HIF1*α* plays an important role in pan-cancer prognosis and immunotherapy, and it may be a novel biomarker with potential prognostic and immunotherapy roles in pan-cancer.

## Figures and Tables

**Table 1:** Details on drugs targeting HIF1*α*.

ID	Name	Characteristic	Action	Number of targets	Examples of effects of targeting HIF1*α*
DB01136	Carvedilol	Nonselective beta-adrenergic antagonist	Modulator	17	Carvedilol downregulates HIF1*α* in the myocardium of volume-overload heart failure [35].
DB02342	2ME2	Angiogenesis inhibitor	Not available	5	2ME2 can aggravate global cerebral ischemia [36] and myocardial ischemia [37].
DB05959	ENMD-1198	A new chemical entity based on a modified chemical structure of 2ME2	Not available	3	ENMD-1198 inhibits HIF1*α* activity, reduces metabolism, induces apoptosis, and destroys microtubules.
DB06082	PX-478	A novel small molecule compound that inhibits the activity of HIF1*α*	Inhibitor	1	PX-478 inhibits the activity of HIF1*α*, which affects the growth of new blood vessels (angiogenesis), the use of glucose for energy, and the prevention of apoptosis (programmed cell death).
DB08687	FG-2216	An active prolyl-hydroxylase inhibitor	Not available	2	FG-2216 can stabilize HIF independent of oxygen availability.

## Data Availability

The datasets analyzed for this study such as patient prognosis, genetic mutations, and pathway enrichment can be found in the GEPIA, TIMER, HPA, GeneMANIA, STRING, SMPDB, cBioPortal, UALCAN, TISDB, and TCGA web resources, and requests to further access to datasets can be directed to zhangdk8616@126.com

## Abbreviations

HIF1*α*: Hypoxia-inducible factor-1*α*
ACC: Adrenocortical carcinoma
BLCA: Bladder urothelial carcinoma
BRCA: Breast invasive carcinoma
CESC: Cervical squamous cell carcinoma
CHOL: Cholangiocarcinoma
COAD: Colon adenocarcinoma
DLBC: Lymphoid neoplasm diffuse large B cell lymphoma
ESCA: Esophageal carcinoma
GBM: Glioblastoma
LGG: Brain lower grade glioma
HNSC: Head and neck squamous cell carcinoma
KICH: Kidney chromophobe
KIRC: Kidney renal clear cell carcinoma
KIRP: Kidney renal papillary cell carcinoma
LAML: Acute myeloid leukemia
LIHC: Liver hepatocellular carcinoma
LUAD: Lung adenocarcinoma
LUSC: Lung squamous cell carcinoma
MESO: Mesothelioma
OV: Ovarian serous cystadenocarcinoma
PAAD: Pancreatic adenocarcinoma
PCPG: Pheochromocytoma and paraganglioma
PRAD: Prostate adenocarcinoma
READ: Rectum adenocarcinoma
SARC: Sarcoma
SKCM: Skin cutaneous melanoma
STAD: Stomach adenocarcinoma
TGCT: Testicular germ cell tumors
THCA: Thyroid carcinoma
THYM: Thymoma
UCEC: Uterine corpus endometrial carcinoma
UCS: Uterine carcinosarcoma
UVM: Uveal melanoma
2ME2: 2-Methoxyestradiol
EPO: Erythropoietin
ENO1: *α*-Enolase 1.

## Supplementary Materials

Supplementary 1 Figure 1: association between HIF1*α* expression and OS in cancer patients.

Supplementary 2 Figure 2: association between HIF1*α* expression and DFS in cancer patients.
