# Supplementary material for: HIF1α: A Novel Biomarker with Potential Prognostic and Immunotherapy in Pan-cancer
Source: Oxid Med Cell Longev. 2022 Jul 8;2022:1246267. doi: 10.1155/2022/1246267 (PMC9289759; doi:10.1155/2022/1246267)
Supplement: Supplementary 2 — Figure 2: association between HIF1α expression and DFS in cancer patients. [file 1246267.f2.pptx]

## Slide 1
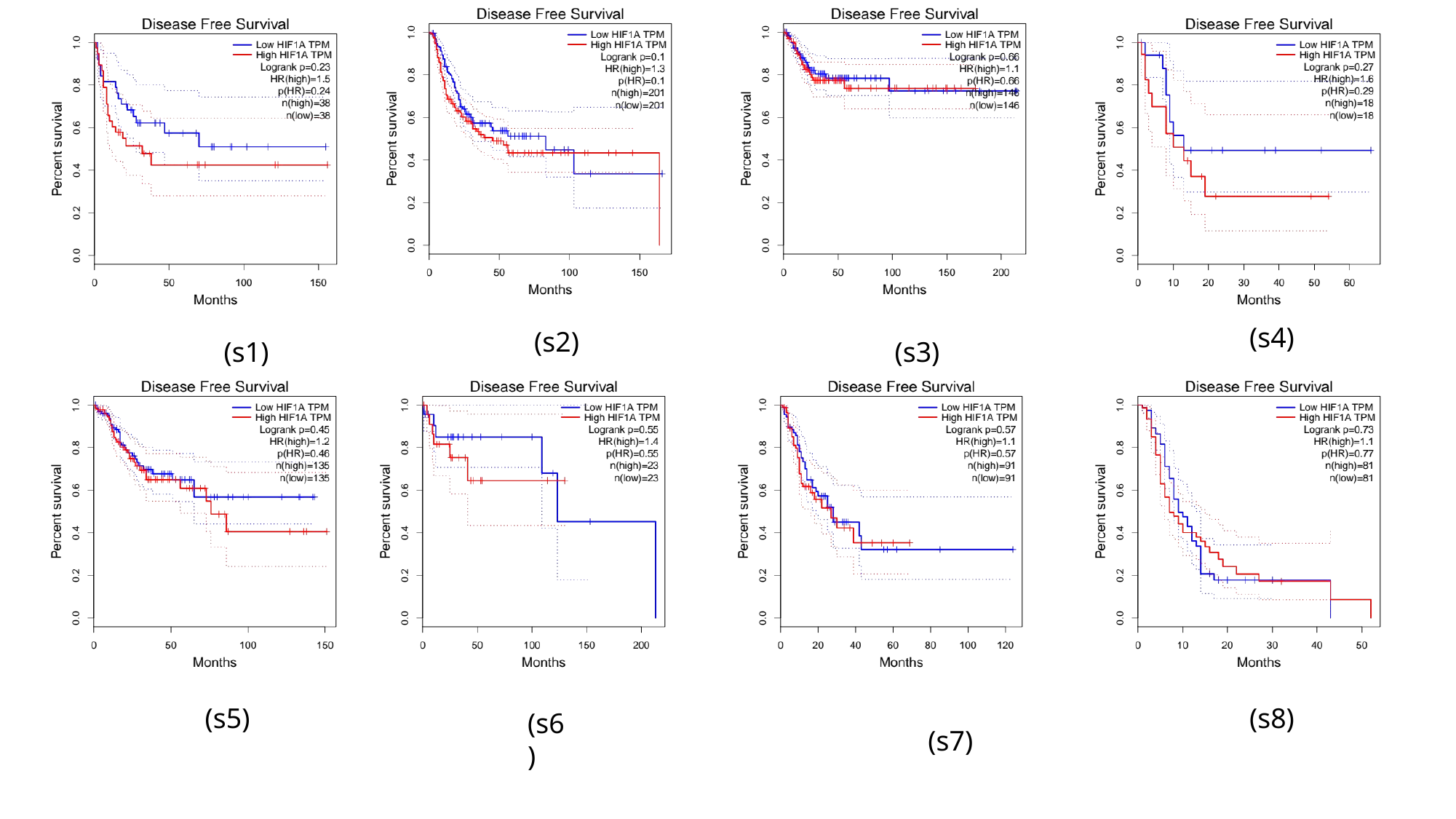

(s4)
(s2)
(s1)
(s3)
(s5)
(s8)
(s6)
(s7)

## Slide 2
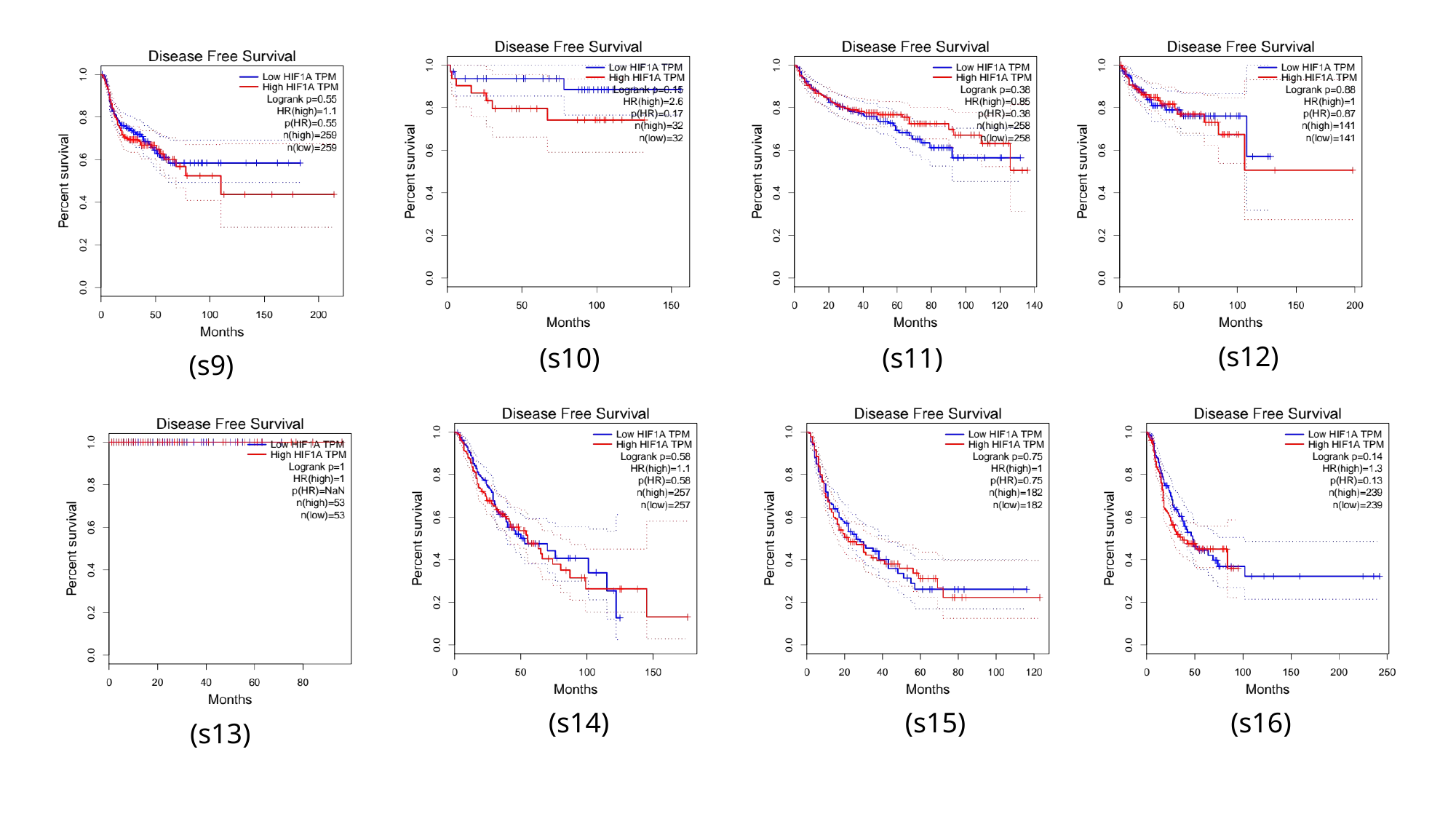

(s12)
(s10)
(s11)
(s9)
(s14)
(s15)
(s16)
(s13)

## Slide 3
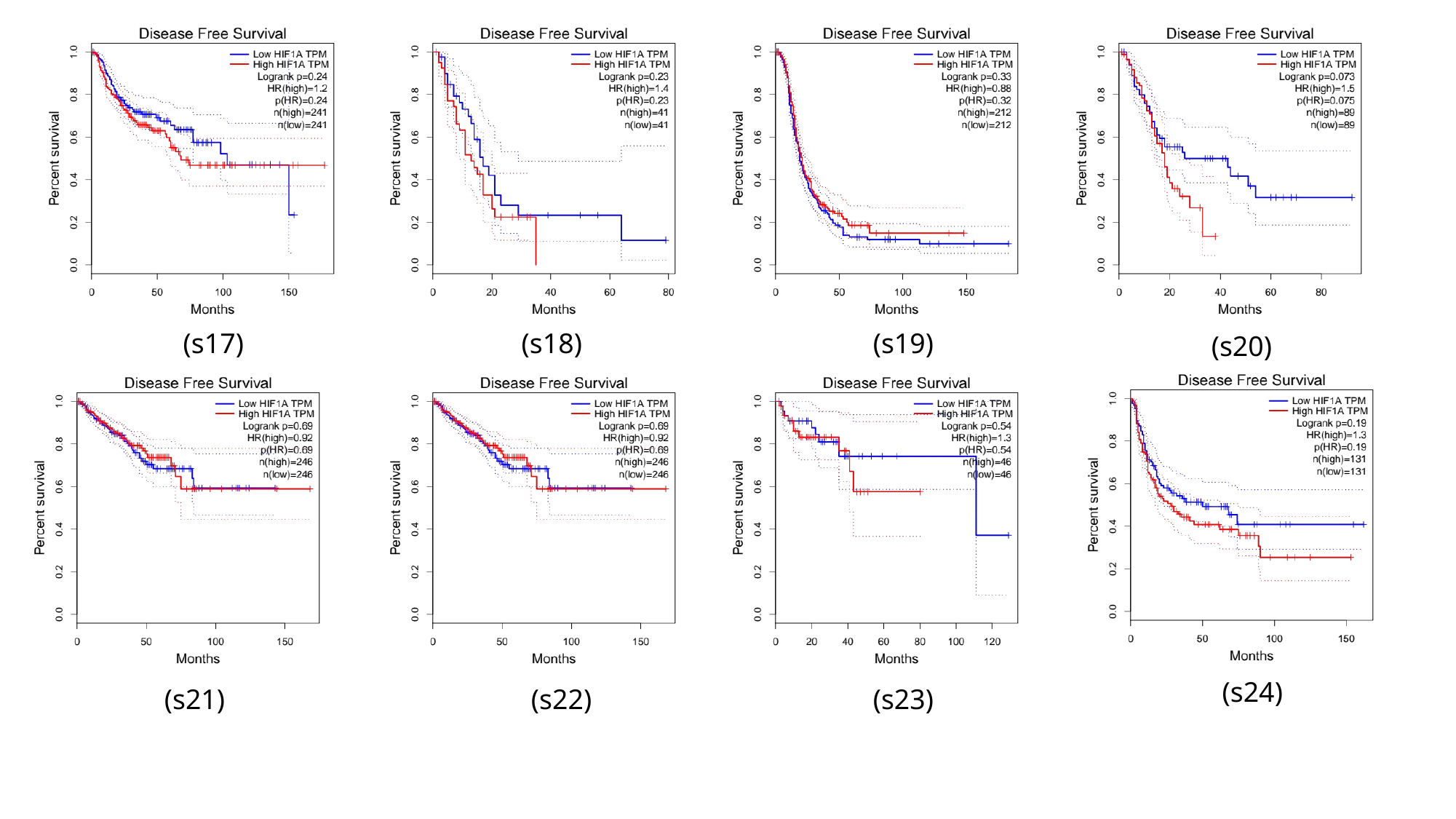

(s17)
(s18)
(s19)
(s20)
(s24)
(s21)
(s22)
(s23)

## Slide 4
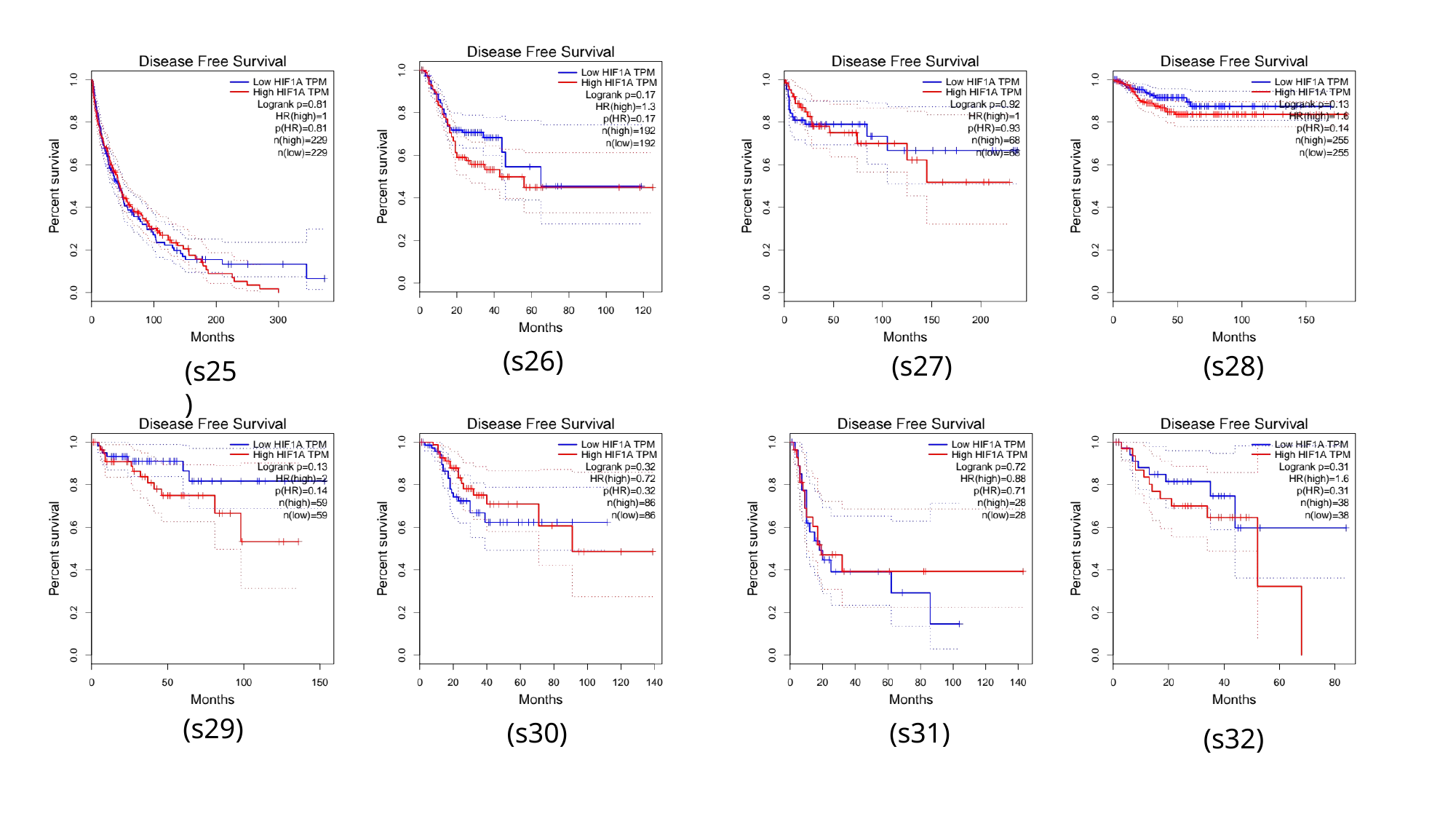

(s26)
(s27)
(s28)
(s25)
(s29)
(s30)
(s31)
(s32)

## Slide 5
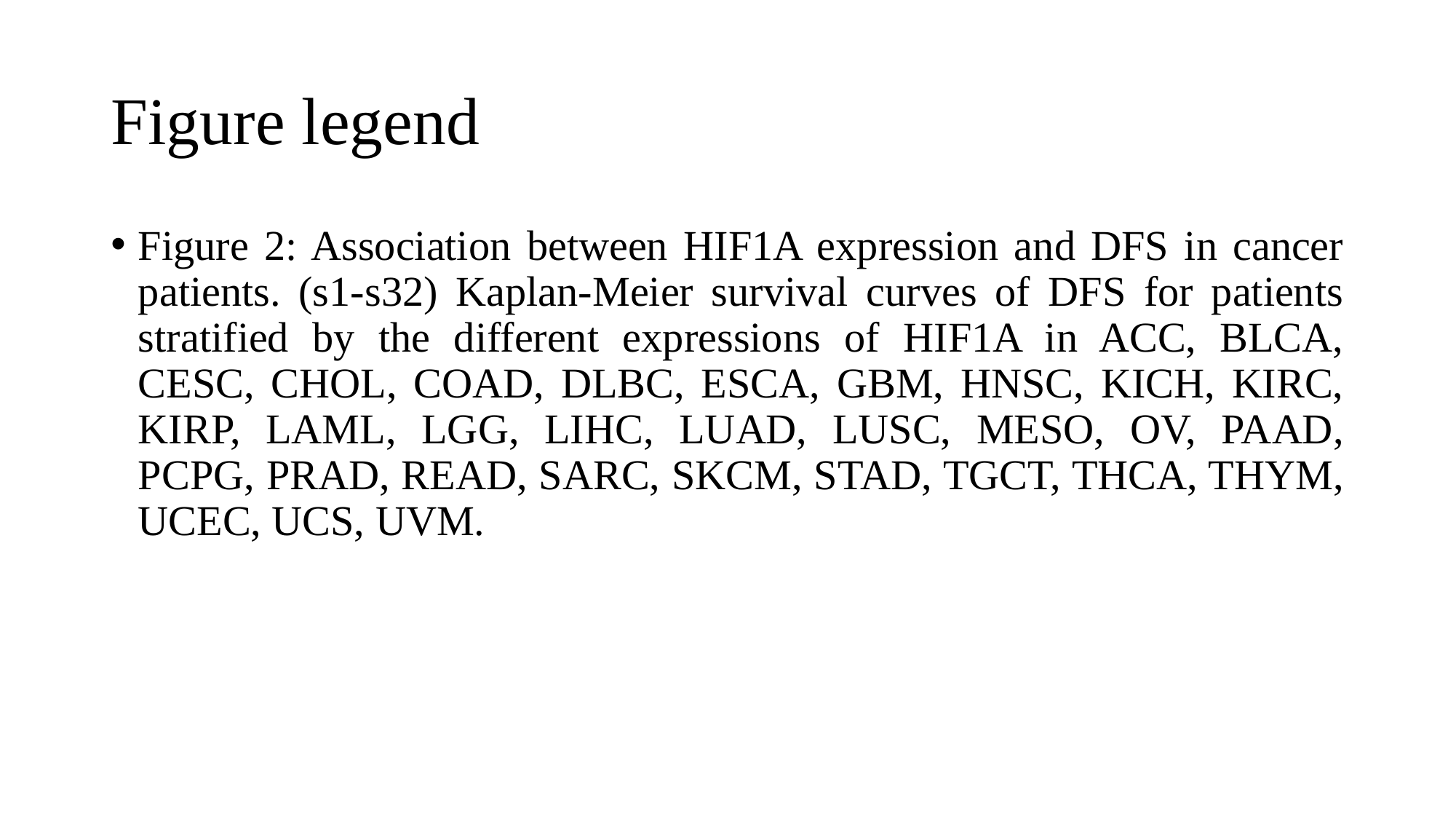

# Figure legend
Figure 2: Association between HIF1A expression and DFS in cancer patients. (s1-s32) Kaplan-Meier survival curves of DFS for patients stratified by the different expressions of HIF1A in ACC, BLCA, CESC, CHOL, COAD, DLBC, ESCA, GBM, HNSC, KICH, KIRC, KIRP, LAML, LGG, LIHC, LUAD, LUSC, MESO, OV, PAAD, PCPG, PRAD, READ, SARC, SKCM, STAD, TGCT, THCA, THYM, UCEC, UCS, UVM.
